# Layup Configuration Effect on Notch Residual Strength in Composite Laminates

**DOI:** 10.3390/ma11020308

**Published:** 2018-02-20

**Authors:** Venkateswaran Santhanakrishnan Balakrishnan, Holger Seidlitz, Sabine Weiß

**Affiliations:** 1 Department of Lightweight Design with Structured Materials, Brandenburg University of Technology Cottbus-Senftenberg, D-03046 Cottbus, Germany; holger.seidlitz@b-tu.de; 2 Chair of Physical Metallurgy and Materials Technology, Brandenburg University of Technology Cottbus-Senftenberg, D-03046 Cottbus, Germany; sabine.weiss@b-tu.de

**Keywords:** composite, notch stress, digital image correlation, Lekhnitskii’s equation

## Abstract

The current trend shows an increasing demand for composites due to their high stiffness to weight ratio and the recent progress in manufacturing and cost reduction of composites. To combine high strength and stiffness in a cost-effective way, composites are often joined with steel or aluminum. However, joining of thermoset composite materials is challenging because circular holes are often used to join them with their metal counterparts. These design based circular holes induce high stress concentration around the hole. The purpose of this paper is to focus on layup configuration and its impact on notch stress distribution. To ensure high quality and uniformity, the holes were machined by a 5 kW continuous wave (cw) CO_2_ laser. The stress distribution was evaluated and compared by using finite element analysis and Lekhnitskii’s equations. For further understanding, the notch strength of the laminates was compared and strain distributions were analyzed using the digital image correlation technique.

## 1. Introduction

Fiber reinforced polymer (FRP) laminates are well known for their excellent stiffness and strength to weight ratio. As a result, FRP are increasingly used to replace metals in numerous fields of engineering where lightweight materials with good mechanical properties are required, such as automotive, aeronautics, astronautics, wind energy, and sports industries.

**Figure 1:**
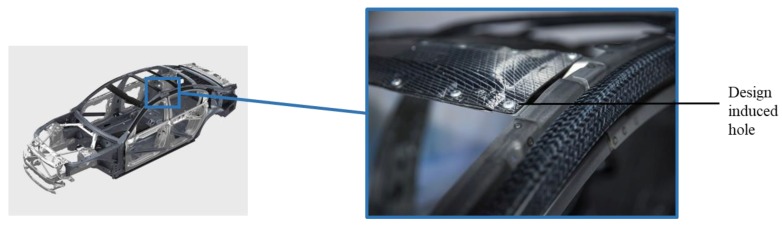
Fiber reinforced polymer (FRP) laminates with design-based holes for joining with steel in the new BMW 7 series (source: BMW, Munich, Germany).

The laminates are often equipped with circular holes in order to join them with other components. Figure 1 shows design-based holes within composite structures used in the BMW 7 series for joining with metals. However, these design-based holes create high stress concentrations close to the notched area due to the interruption of the force flux in the fiber direction. These stress concentrations are often failure initiation regions in structural parts. Due to their good mechanical performance, FRP composites are increasingly used in the design of structural parts. Therefore, intensive study on the fatigue behavior of notched FRP laminates is [1,2,3,4] required. Many researchers have studied the effect of holes within FRP composites [5,6] but only a few with regard to the layup arrangement and its effects on the strength of the material.

Woven fabric composites have gained their importance due to their bidirectional reinforcement in a single layer of fabric which provides more balanced properties in the fabric plane than unidirectional laminae. As well as their excellent drapability over complex geometries, low manufacturing costs and impact resistance quantify them as preferred structural materials. Thermoset composites offer excellent dimensional stability, receptiveness to paint, a lower thermal expansion coefficient compared to steel, high impact strength, while relatively low weights quantify them as functional components for automotive, aeronautics, astronautics, and in wind energy.

Many authors have investigated the performance on the notch stress concentration in FRP laminates. In ref. [7], it was identified that there is 30% strength reduction in notched composite, compared with unnotched composite. In ref. [8], the Waddoups, Eisenmann and Kaminski (WEK) model assumes that there is a high stress concentration around the hole in the FRP composite. In ref. [1,2,4,5,6,7,8], many authors identified experimentally and numerically there is a high stress concentration around the edge of a hole. In ref. [9,10], notched stress on quasi-isotropic FRP laminates were performed and it was found that there is a development of an intense damaged zone near the hole edge. The presence of this stress concentration in a notched composite causes a wide range of damage and a failure mechanism when compared with an unnotched composite specimen. Thus, there is a necessity to identify the extensive full-field strain data to identify the failure behavior, rather than the limited experimental strength reduction values. However, very few studies have been made to identify the failure behavior using full-field strain data. In addition, there have been limited studies that explained the layup sequence and its influence in notch stress concentration. Thus, the aim of this paper is to analyze the strain development with respect to various layup architectures in notched glass/epoxy woven composites. The notch strength of the four woven fabric FRP laminates ^f^[0°]_4_, ^f^[45,0°]_S_, ^f^[0,45°]_S_ and ^f^[0°]_3_ was investigated. By using Lekhnitskii’s equation and finite element analysis, the stress concentration was analyzed. For further understanding, digital image correlation (DIC) technique was employed to obtain full-field surface strain measurements in differently notched laminates under tensile loading. 

## 2. Material

**Figure 2:**
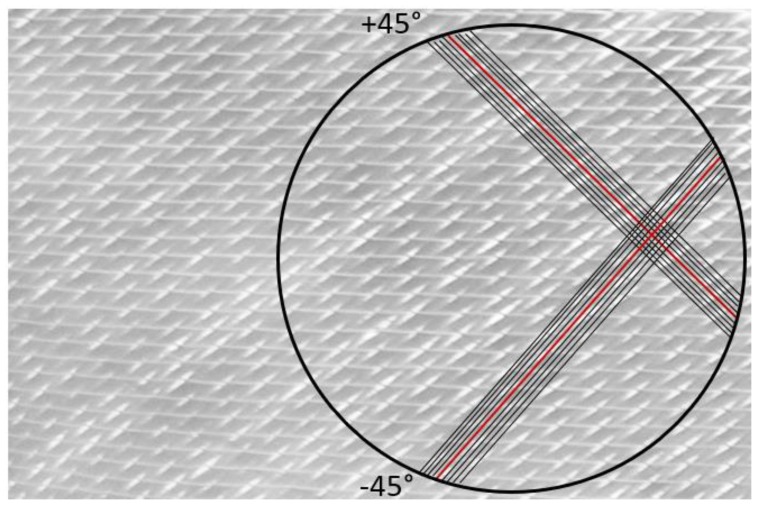
Glass non-crimp fabric 220 g/m² (biaxial, silane) fabric.

Commercially available 220 g/m² non-crimp E-glass biaxial reinforcement and standard epoxy resin were used as matrix material for the FRP. The construction of biaxial non-crimp fabric (–45°/+45°) is shown in Figure 2. ^f^[0°]_4_, ^f^[45,0°]_S_, ^f^[0,45°]_S_, and ^f^[0°]_3_ laminates were manufactured using the vacuum assisted resin transfer molding (VARTM) process. The VARTM process was preferred because it allows the use of low-cost tooling for the production of high quality composite parts. For curing, the samples were kept under vacuum at ambient temperature of 24 °C for 24 h.

**Figure 3:**
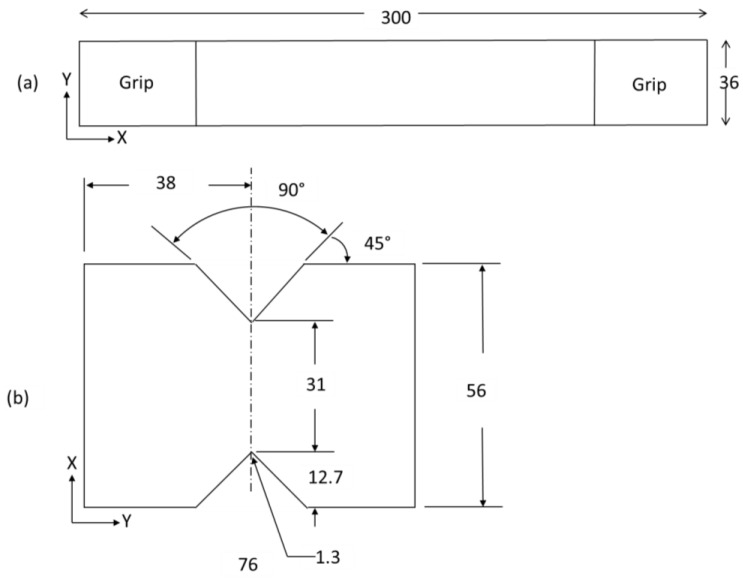
Specimen geometry (dimension in mm): (**a**) tensile specimen and (**b**) shear specimen.

Plane strain ($\varepsilon_{11},\;\varepsilon_{22}$) was calculated by performing tensile fatigue tests on non-notched specimens according to the standard ASTM D3039 [11]. Elastic modulus (*E*_11_ and *E*_22_) results presented in this section were obtained by testing five specimens for each layup and standard deviation (SD) between the samples is compiled in Table 1. Since the woven fabric was used to make FRP laminates, laminates were considered to be transversely isotropic. Thus, the elastic moduli in the *x*–*y* plane directions are equal (*E*_11_ = *E*_22_) and the laminate will only have five independent elastic constants in the elasticity tensor. Shear modulus was calculated experimentally using the V-Notched Rail Shear Method according to the standard ASTM D 7078/D 7078M–05 [12]. The dimensions used for tensile and shear tests, respectively, are shown in Figure 3.

The tests were carried out at room temperature with a linear torsion all-electric dynamic test instrument (Instron ElectroPuls E10000, Darmstadt, Germany). The crosshead speed was maintained at 2 mm/min, shear modulus (*G*_12_), uniform slope between shear stress and shear strain (*G*_12_ = Δτ/Δε). Engineering shear strain ε and shear stress τ are calculated using Equation (1).

(1) $$\varepsilon =\left|\varepsilon_{+45}\right|+\left|\varepsilon_{-45}\right|;\;\tau =P/A$$

where *A* is the area of the specimen and $\varepsilon_{+45},\;\varepsilon_{-45}$ are measured by digital image correlation (DIC) technique and the technique is explained in Section 3.4. GOM Correlate Evaluation Software was utilized for post processing. Three specimens were tested in the V-Notched Rail Shear Method and all the specimens were spray painted and a video was recorded. The average maximum force of three specimens was calculated. To reduce the post processing experiments, the specimen which had a close value to the average force was used for post processing. The corresponding shear force value (*P*) was identified from the experimental data. By measuring the uniform slope between shear stress and shear strain, the shear modulus (*G*_12_) was calculated and is listed in the Table 1. 

## 3 Methods

### 3.1. Preliminary Study on the Quality of Circular Holes

A preliminary study was conducted with quasi isotropic ([45°, –45°, 0°, 90°]_S_) carbon-epoxy composite laminate to choose the best method to produce circular holes with good quality in FRP laminates [2]. The holes were produced by drilling processes or laser methods. Introduction of the holes by cw-CO_2_ lasers caused a reduction in maximum force of 27.94% compared to the unnotched specimen and drilling results a reduction of 26.06%, respectively. So, there were only slightly improved characteristic values obtained for drilling compared to cw-CO_2_ laser process. The laser system can offer high cutting speeds, better flexibility, less tool wear, and less noise and dust compared to drilling. The laser system allows a high degree of automation compared to the milling machine because changing the drill bit for various hole diameters increases the processing time. In addition, tool wear increases delamination during drilling.

**Figure 4:**
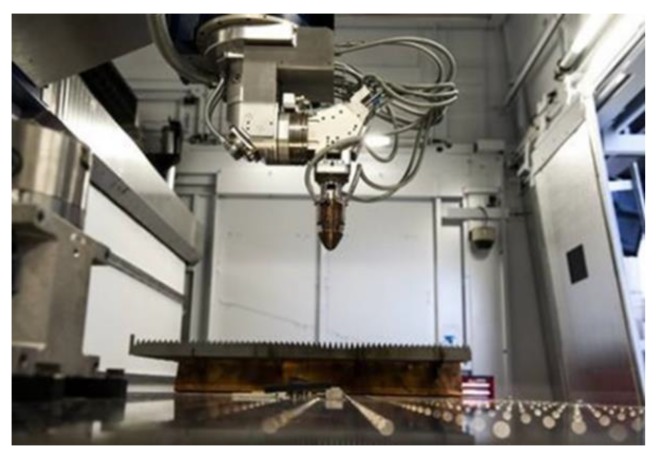
Set-up of 3D robot assisted TRUMPF TruLaser Cell 7040 5 kW cw-CO_2_ laser system.

Hence a 3D robot assisted TRUMPF TruLaser Cell 7040 5 kW cw-CO_2_ laser system was used for hole machining in this study (Figure 4).

### 3.2. Lekhnitskii’s Equation

**Figure 5:**
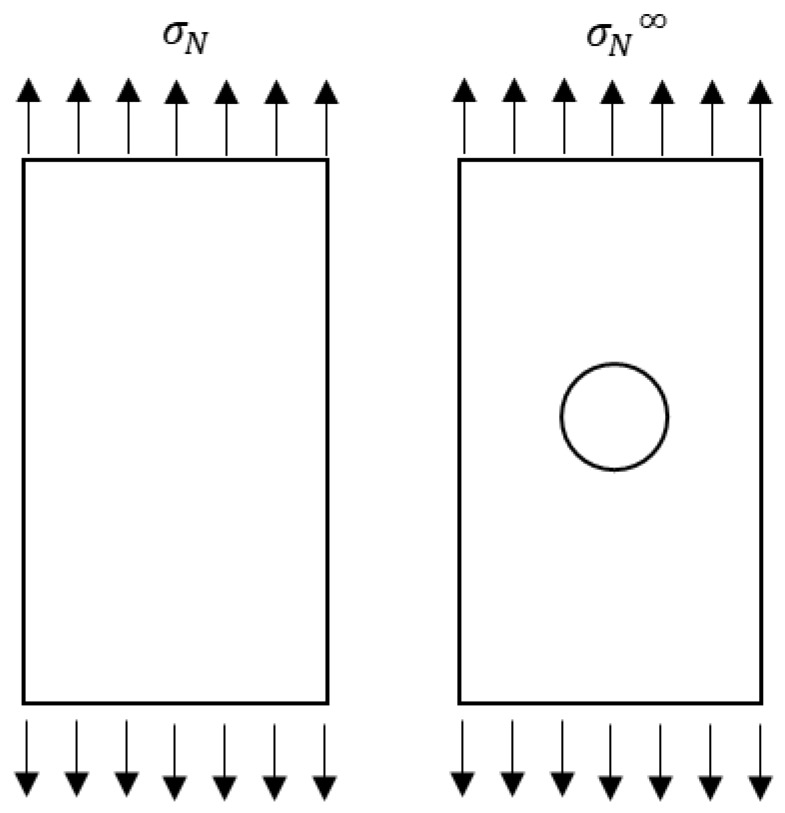
Applied stress within the crack regions of unnotched as well as notched samples.

The strength of the laminates ($\sigma_{N}{}^{\infty }/\sigma_{O}$) was calculated for various layup configurations with varying hole diameters (4, 6, and 8 mm). The infinite width notch tensile strength $\sigma_{N}{}^{\infty }$ is calculated by multiplying the finite width notch tensile strength ($\sigma_{N})$ by the finite width correction factor ($K_{T}{}^{\infty }/K_{T})$. $\sigma_{O}$ is the tensile strength of the corresponding laminates [13,14] without notch. Infinite width notch tensile strength and tensile strength of the unnotched laminates in the failure region are explained in Figure 5 and the corresponding values are calculated using the Lekhnitskii’s equation. 

For an infinite width orthotropic plate containing a circular hole of radius *R* subjected to a uniform stress $\sigma^{\infty }$ in the y direction, the normal stress $\sigma_{y}$, along the *x*-axis in front of the hole can be approximated by Equation (2) [7,15,16]. Please check all the variables mentioned in the main text. The format (italic or not) should be unified through the main text.

(2) $$\sigma_{y}\left(x,0\right)=\frac{\sigma^{\infty }}{2}\{2+{\left(\frac{R}{x}\right)}^{2}+3{\left(\frac{R}{x}\right)}^{4}-\left(K_{T}{}^{\infty }-3\right)\left[5{\left(\frac{R}{x}\right)}^{6}-7{\left(\frac{R}{x}\right)}^{8}\right]\};\;x>R$$

where $K_{T}{}^{\infty }$ is the orthotropic stress concentration factor for a circular hole in an infinite width plate as expressed by Equation (2).

(3) $$K_{T}{}^{\infty }=1+\sqrt{2\left(\sqrt{E_{xx}/E_{yy}}-\vartheta_{xy}\right)+\left(E_{xx}/G_{xy}\right)}\;$$

$E_{xx}$, $E_{yy},$ and $G_{xy}$ are axial, transversal, and shear modules respectively. $\vartheta_{xy}$ is the major Poisson’s ratio. To modify Equation (1) for a finite width plate, a correction factor is implemented and for the composite laminates, equivalent modules for the laminates are replaced. The finite width correction (FWC) factor for orthotropic laminates with a circular hole is given in Equation (3). $K_{T}$ and $K_{T}{}^{\infty }$ are the stress concentration factors for finite and infinite width orthotropic plates, respectively.

(4) $$\frac{K_{T}{}^{\infty }}{K_{T}}=\frac{3\left(1-D/W\right)}{2+{\left(1-D/W\right)}^{3}}+\frac{1}{2}{\left(\left(D/W\right)M\right)}^{6}\left(K_{T}{}^{\infty }-3\right)\;X\;\left[1-{\left(\left(D/W\right)M\right)}^{2}\right]$$

(5) $$M^{2}=\frac{-1+\sqrt{1-8\left[\frac{3\left(1-D/W\right)}{2+{\left(1-D/W\right)}^{3}}-1\right]}}{2{\left(D/W\right)}^{3}}$$

Equation (5) shows the sample dimension related expression to calculate *M*, where *D* (=2*R*) is the diameter of the holes, *W* the width of the sample, and $K_{T}$ the orthotropic stress concentration factor for a circular hole in a finite width plate.

### 3.3. Finite Element Analysis (FEA)

FEA can improve the understanding of structural behavior and local failure mechanisms due to notches inside the plate. The normal stress distribution (NSD) depending on the geometry of the hole was executed using the commercially available ANSYS R18.1 software. NSD is the ratio of normal stress $\sigma_{y}\left(x=0\right)$ along the x-axis in front of the hole to the stress applied on the laminate [17,18]. NSD was calculated using FEA and the results were compared with the values obtained from Lekhnitskii’s equation. This helps to understand damage distribution and evolution of stress at the notch edge.

### 3.4. Digital Image Correlation (DIC)

DIC technique is a full field non-contact optical technique used to measure the surface deformation. DIC registers two (or more) images of the same structure subjected to various loadings (e.g., mechanical loading or thermal loading) to obtain full-field surface strain measurements of the entire specimen [17,19,20,21]. Optical full-field measurement techniques are increasingly used in material testing, fracture mechanics, and vibration analysis. Samples are coated with black/white spray paints to create a random speckle pattern to obtain suitable surface contrast for DIC. Videos of the deformation were recorded with a digital single-lens reflex camera and the videos were processed with GOM Correlate Evaluation Software (2017) to analyze the strain distribution.

## 4. Results and Discussion

### 4.1. Strength of the Laminates

The strength of the laminates ($\sigma_{N}{}^{\infty }/\sigma_{O}$) was calculated as explained in Section 3.2. The strength of the notched glass/epoxy laminates with different layup configurations are listed in Table 2.

All tensile tests were carried out according to ASTM D 5766/D 5766M standard [22]. For all calculations, the width of the laminates (*W*) was maintained at 36 mm and three different sizes of the hole (2*R*), 2*R*/*W* ratio were varied uniformly in all laminates.

**Figure 6:**
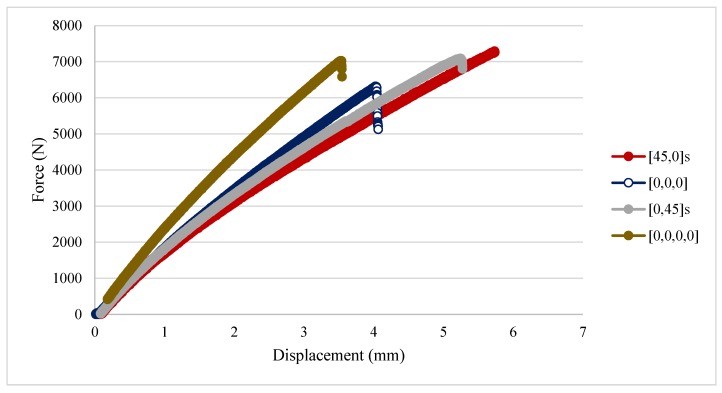
Force-displacement curve of unnotched samples.

Representative force-displacement curves of the unnotched laminates are shown in Figure 6. The results show that mixing the layup orientation (^f^[0,45°]_S_ and ^f^[45,0°]_S_) result in a reduction in elastic modulus but at the same time in an improvement in the strength of the material.

**Figure 7:**
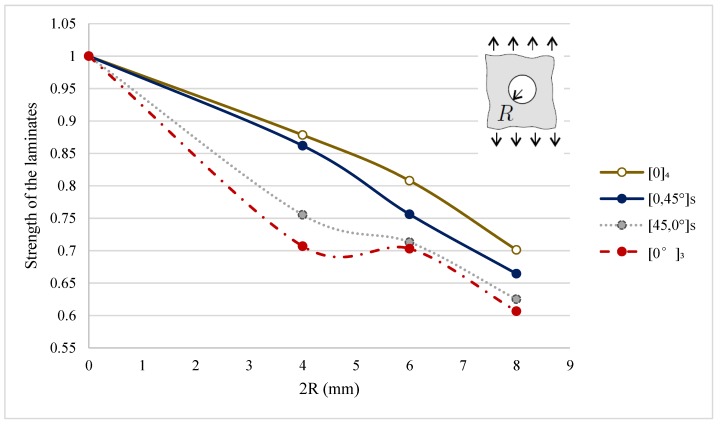
Strength of the laminates ($\sigma_{N}{}^{\infty }/\sigma_{O}$) vs. size of the circular hole (2*R*) in the center of the laminates.

Figure 7 explains the hole size effect for the various laminates. Thus, when a laminate with a hole is subjected to tension, there is high stress concentration inside the laminate. Since, more fibers are destroyed by the large hole, stress concentration increases with increasing size of the hole. The stress gradient in a small hole can be distributed better compared to that in larger holes. It is also evident that there is a big difference between the strength plots of different layup arrangements. The most interesting outcome of the experiment is that the laminates [0,45]_S_ and [45,0]_S_ almost have the same strength values in the unnotched case ([45,0]_S_: 7270N and [0,45]_S_: 7055N) but with a circular hole in the center the strength was reduced by 25% for [45,0]_S_ but only by 14% for [0,45]_S_. This unbalanced strength decrease continues with larger hole size, too. Biaxial [45,0]_S_ laminate consists of 2nd and 3rd lamina at an acute angle to the pulling direction during the experiment whereas biaxial [0,45]_S_ laminate has parallel fiber orientation at the 2nd and 3rd lamina. Ply at the inner end plays a vital role in strength determination of notched laminate but there is no actual theory to support the correlation between layup and strength reduction for a notch problem. In unnotched unsymmetrical laminate shear extension and bending-stretching are coupled, therefore high reduction in the strength of the laminate will occur if any hole is introduced in unsymmetric laminates. The [0]_4_ laminate has the best outcome compared to other laminate layup architectures.

### 4.2. Normal Stress Distribution 

Finite element analysis was conducted under static structural analysis. A four noded element with six degrees of freedom at each node (SHELL181) was used because it allows the layered section with different orientations to be defined. A rectangular plate with a circular hole in the center as shown in Figure 1 was taken for calculating stresses induced by the applied load. The values of in-plane Young’s moduli (i.e., *E*_1_ and *E*_2_), Poisson’s ratio ($\upsilon_{12}$), and shear moduli (*G*_12_) were calculated using the experimental results and are shown in Table 1. The current investigation is not sensitive to the values in thickness direction (values are considered as identical in the 90° direction, $\upsilon_{12}=\upsilon_{13}$ and *G*_12_ = *G*_13_). A plate with identical dimension asin the experiments was used in FEA. A typical finite element coarse mesh division was used to model the laminates.

**Figure 8:**
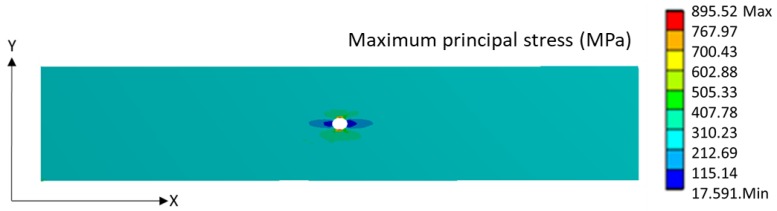
Maximum principal stress distribution for 4 mm notched ^f^[0°]_4_ laminate.

To measure the stress along the x-axis in front of the hole, a path geometry was constructed to obtain the stress along that path. The calculated values were divided by the applied stress to calculate the NSD. The effect of a hole in the maximum principal stress distribution for a 4mm notched ^f^[0°]_4_ laminate is shown in Figure 8. There is a substantial stress concentration at the hole edges in the X–Y direction which drops low at a certain distance from the hole.

**Figure 9:**
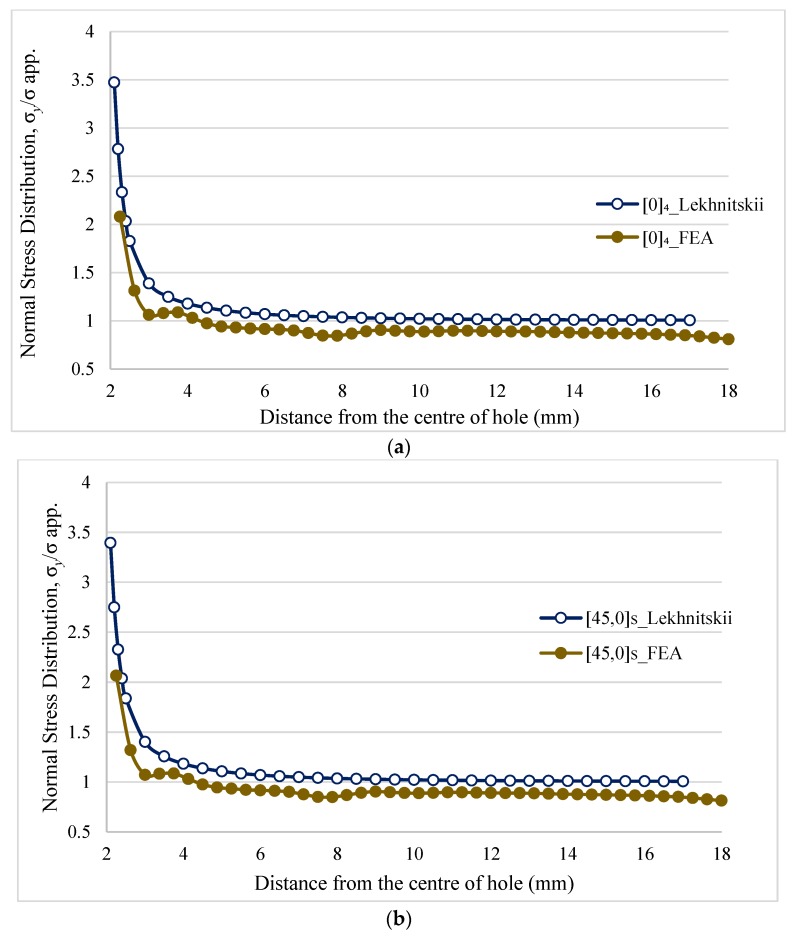

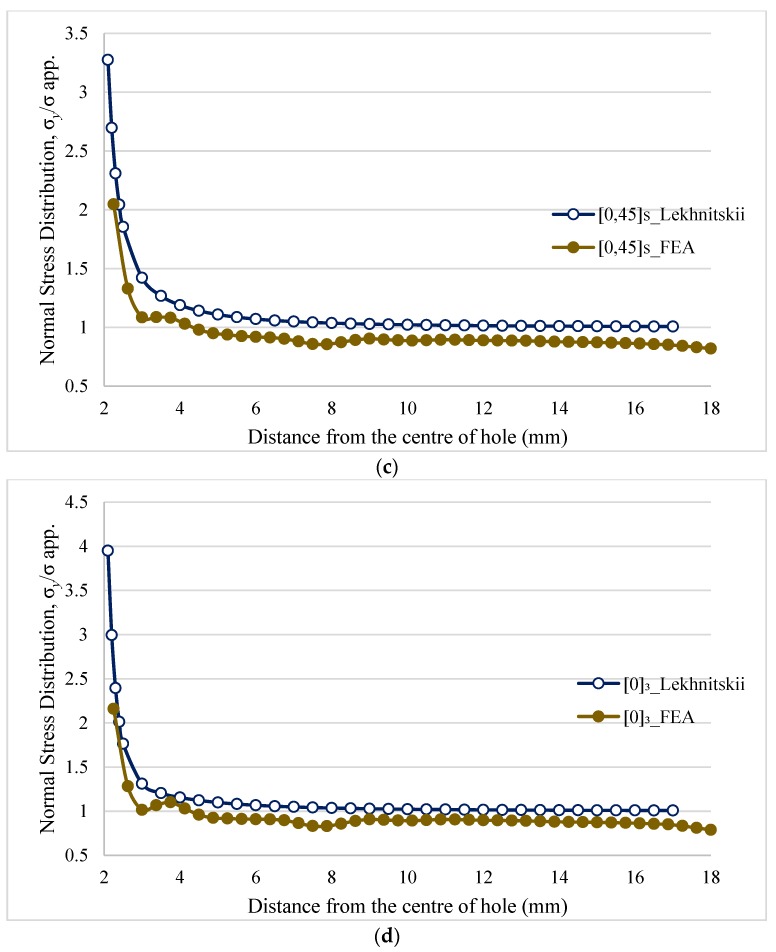
(**a**) Normal stress distribution vs. distance from the center of the hole in 4 mm notched ^f^[0°]_4_ laminate; (**b**) Normal stress distribution vs. distance from the center of the hole in 4 mm notched ^f^[45,0°]_S_ laminate; (**c**) Normal stress distribution vs. distance from the center of the hole in 4 mm notched ^f^[0,45°]_S_ laminate; (**d**) Normal stress distribution vs. distance from the center of the hole in 4 mm notched ^f^[0°]_3_ laminate.

The results of Lekhnitskii could be validated with the results of FEA by calculating the stress concentration around the circular hole with respect to various layup arrangements. The results will give in-depth understanding of the stress concentration. As shown in Figure 9, Lekhnitskii and FEA results have slight variations at the hole edges from 2.1 mm to 2.25 mm but in sequel both graphs are similar. This behavior is obvious in all these graphs. According to both techniques, the highest stress occurs at the edge of the hole. Similar to Section 3, in unsymmetric laminate [0]_3_ the highest stress concentration occurs compared to all unsymmetric laminates. As explained in Section 3 there is a very high strength difference between the laminates [45,0]_S_ and [0,45]_S_, therefore these two laminates were compared in more detail. As expected, [0,45]_S_ laminate (strength reduction −14%) has low stress concentration compared to [45,0]_S_ laminate (strength reduction −25%). But the difference in NSD was not proportional to the strength reduction. Further investigations on this strength reduction were required for a proper understanding, so digital image correlation (DIC) technique was used to analyze the damage mechanisms inside the laminates.

### 4.3. Strain Evolution

**Figure 10:**
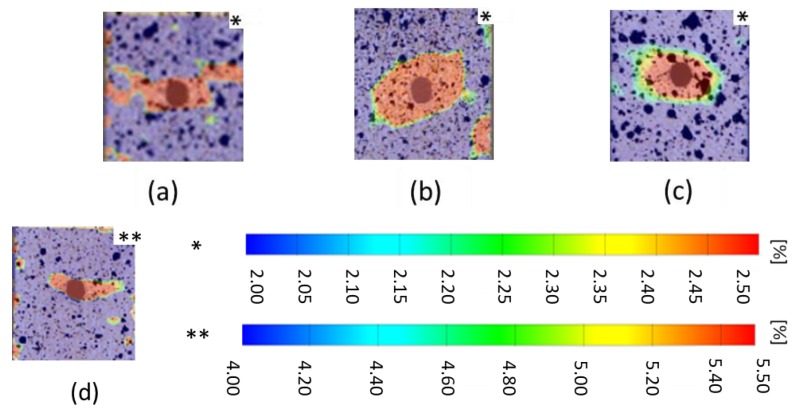
Longitudinal strain ($\varepsilon_{22}$) field on the 4 mm notched laminate due to tensile loading. (**a**) ^f^[0°]_4_ laminate; (**b**) ^f^[0°]_3_ laminate; (**c)** ^f^[45,0°]_S_ laminate; and (**d**) ^f^[0,45°]_S_ laminate.

As explained in Section 3.4, all samples were prepared with a random speckle pattern using a black/white spray and the longitudinal strain ($\varepsilon_{22}$) field close to the notch was calculated from the DIC contrast. The strain fields were compared with the ones of other laminates. All strains were calculated around 1% prior to the ultimate failure of the laminates. In DIC, the subset size is a user defined parameter, which is critical for the measured accuracy of the samples [14]. To have a uniform result in all samples, a uniform subset size of 19 × 16 pixels was used for processing. As Figure 10 shows, the [45,0]_S_ laminate (strength reduction −25%) has the highest strain concentration of all laminates. The strain within this laminate is in the range of 4%–5.5%, whereas in all other laminates strains in the range of 2%–2.5% occur. This could be the reason for the higher strength reduction in the laminate [45,0]_S_. In unsymmetric laminate, even though it has a lower strain range compared to [45,0]_S_, the strain is significantly higher than in all other laminates. So, this could be the reason for the lowest strength of this laminate compared to the symmetric laminates.

## 5. Conclusions

The effect of notch stress concentration in glass/epoxy woven composite with four different layup arrangements was investigated and compared. Analytical, numerical, and experimental test investigations were performed and analyzed. The results confirmed that the layup architecture has a significant influence on the notch strength. The following behaviors were observed in this study,

- Tensile test on notched and unnotched [45,0]_S_ and [0,45]_S_ laminates revealed, even though they have similar unnotched strength ([45,0]_S_: 7270N and [0,45]_S_: 7055N), when they are subjected to circular hole, strength was reduced by 25% for [45,0]_S_ but only 14% for [0,45]_S_.
- In full-field strain measurement, [45,0]_S_ shows a high longitudinal strain (4%–5.5%) around the notch compared to [0,45]_S_ laminate with 2%–2.5%.
- Unsymmetric laminate shows high stress distribution at the edge of the hole compared to symmetric laminates.
- All laminates show very high stress concentration near the edge of hole. Stress distribution is linear after 2 mm from the edge of hole. Until 2 mm, normal stress distribution increases exponentially towards the edge of the hole.

## Figures and Tables

**Table 1:** Experimentally determined properties of the laminates.

Layup	*E*_11_ = *E*_22_ (All Sample) (GPa)	*E*_11_ = *E*_22_ (Average) (GPa)	SD (*E*_11_) (GPa)	ʋ_12_	*G*_22_ (GPa)	Thickness (mm)
^f^[0°]_4_	17,783.2 17,394.4 17,508.0 17,433.7 17,360.7	17.495	0.1696	0.139	1.597	0.66
^f^[0,45°]_S_	12,015.8 12,247.5 11,655.5 12,203.5 11,834.6	11.992	0.2492	0.306	1.388	0.66
^f^[45,0°]_S_	10,656.8 10,007.8 11,574.2 10,154.1 10,300.4	10.538	0.6270	0.169	1.062	0.66
^f^[0°]_3_	16,196.5 16,478.4 16,763.9 15,900.4 16,787.5	16.425	0.3797	0.05	0.892	0.54

**Table 2:** Notched strength test results for various glass/epoxy layups.

Layup	2*R* (mm)	2*R*/*W*	$\sigma_{N}$ (MPa)	$\sigma_{N}{}^{\infty }$ (MPa)	$\sigma_{N}{}^{\infty }/\sigma_{N}$
^f^[0]_4_	4	0.111	342.13	346.69	0.8784
^f^[0]_4_	6	0.167	313.78	318.87	0.8079
^f^[0]_4_	8	0.222	261.58	276.75	0.7012
^f^[0,45°]_S_	4	0.111	293.30	297.21	0.8619
^f^[0,45°]_S_	6	0.167	255.62	260.74	0.7561
^f^[0,45°]_S_	8	0.222	216.52	229.11	0.6644
^f^[45,0°]_S_	4	0.111	264.90	268.43	0.7553
^f^[45,0°]_S_	6	0.167	249.02	253.43	0.7131
^f^[45,0°]_S_	8	0.222	209.97	222.16	0.6251
^f^[0]_3_	4	0.111	262.84	266.34	0.7069
^f^[0]_3_	6	0.167	263.02	264.91	0.7031
^f^[0]_3_	8	0.222	216.18	228.61	0.6067
